# 

**Published:** 1843-11-25

**Authors:** 


					﻿TO SUBSCRIBERS.
The attention of our subscribers, who are in arrears, is respectfully invited to the terms of
subscription. An early attention to our claims is solicited. When it is considered that the
Examiner is purely a professional journal, and unsupported by the capital of a publishing
house, the propriety of a compliance with our demands will be generally recognized. Funds
current in the state where the subscriber resides, will be received.
THE MEDICAL· EXAMINEE, AND RETROSPECT OF THE MEDICAL SCIENCES,
IS PUBLISHED EVERY ALTERNATE SATURDAY.
OFFICE AT J. C. TURNPENNY’S, N. E. CORNER OF SPRUCE AND TENTH STREETS.
Subscription Price, Three Dollars a year, invariably in advance. Any person enclosing
Ten Dollars on a solvent bank, free of postage, will receive five copies.
Communications and Letters to be addressed (always pre-paid) in every instance to the Editor of
the Medical Examiner, Philadelphia.
Postmaster’s franks can always be obtained for remittances.
This Journal is subject to newspaper postage only.
Rates of Advertising. For first insertion of 20 lines or less, $1—each additional insertion 50 cts.
For advertisements occupying a whole page, and inserted yearly or half-yearly, a large reduction from
the above rates will be made.
—_____________________________ MERRIHEW AND THOMPSON, PRINTERS, 7 C ARTER’s ALLEY.
				

